# Nuke-sparing regimens as a main simplification strategy and high level of toxicity resolution after antiretroviral switch: the SWITCHART Study

**DOI:** 10.7448/IAS.17.4.19819

**Published:** 2014-11-02

**Authors:** Ana Carrero-Gras, Antonio Antela, Jessica Muñoz-Rodríguez, Marta Díaz-Menéndez, Pompeyo Viciana, Adriadna Torrella-Domingo, José Sanz-Moreno, María Jesús Téllez-Molina, Javier Moreno, José Hernández-Quero, Isabel A Pérez-Hernández, Pere Domingo-Pedrol

**Affiliations:** 1 Infectious Diseases-HIV Unit, Hospital General Universitario Gregorio Marañón, Madrid, Spain; 2 Infectious Diseases Unit-Internal Medicine Department, Hospital Clínico Universitario de Santiago, Santiago de Compostela, Spain; 3 Internal Medicine Department, Hospital Universitario de la Santa Creu i Sant Pau, Barcelona, Spain; 4 Internal Medicine Department, Hospital Universitario La Paz, Madrid, Spain; 5 Internal Medicine Department, Hospital Universitario Virgen del Rocío, Seville, Spain; 6 Internal Medicine Department, Hospital Universitario Vall d'Hebron, Barcelona, Spain; 7 Internal Medicine Department, Hospital Universitario Príncipe de Asturias, Alcalá de Henares, Spain; 8 Internal Medicine Department, Hospital Universitario Clínico San Carlos, Madrid, Spain; 9 Internal Medicine Department, Hospital Universitario Miguel Servet, Zaragoza, Spain; 10 Internal Medicine Department, Hospital Universitario San Cecilio, Granada, Spain; 11 Clinical Management of Infectious Diseases Unit, Hospital Universitario Virgen de la Victoria, Málaga, Spain

## Abstract

**Background:**

The advent of combined antiretroviral therapy (ART) in the past decade has led to HIV suppression in most cases. Virological failure was the main reason for ART switch a few years ago; however, toxicity and treatment simplification have now gained importance due to the availability of more effective and convenient drugs. This study assessed the reasons for ART switch in daily practice.

**Material and Methods:**

Observational retrospective study that included patients whose ART was switched between January 2011 and July 2012. Patients with any other switch during the follow-up period (until September 2013) were excluded.

**Results:**

A total of 246 patients were included. Main reasons for ART switch were simplification (33%) and toxicity (31%), followed by clinical trial inclusion (13%), virological failure (6%), drug interaction (4%), patient decision (3%), lack of adherence (2%), pregnancy (1%) and other (8%). Eighty patients switched to a simpler regimen (median age 48 [40–53], mean CD4 count 608±265 cells/cl, 89% <50 copies/ml, mean number of previous regimens 6±5, mean time on previous ART 3±2 years). In this case, previous ART mostly included 2NRTI+1PI/r (54%) (Figure 1). The simplification strategy mainly contained nuke-sparing regimens (60%) based on PI (DRV/r 48%): monotherapy 46%, dual therapy 13% (PI/r+maraviroc 9%, PI/r+NNRTI 4%) and triple therapy 1% (PI/r+maraviroc+raltegravir). The second preferred simplification option was 2NRTI+1NNRTI (24%). Seventy-seven patients switched due to toxicities (median age 47 [43–53], mean CD4 count 606±350 cells/μl, <50 copies/ml 82%, mean number of previous regimens 4±3, mean time on previous ART 3±3 years). Renal (25%) and CNS (18%) toxicities were the main reasons for ART switch, followed by diarrhoea (16%), liver enzyme elevation (ALT 10%; AST 9%; bilirubin 7%), lipid elevation (cholesterol 5%; triglycerides 8%), nausea (7%) and other (=5%) (Figure 2). All patients with renal toxicity were under TDF and in most cases this drug was removed from the new regimen (with 3TC/ABC or nuke-sparing). Among patients with CNS toxicity, 79% were taking EFV; the main new treatment was a second-generation NNRTI (ETR)+2NRTI. Toxicities were completely resolved in 66% of patients, partially resolved in 22% and not resolved in only 12%; the median time from ART switch to toxicity resolution was 4 (2–8) months.

**Conclusions:**

The main reasons for ART switch in daily practice are simplification and toxicities, renal and CNS toxicities being the most prevalent. The preferred simplification strategies are nuke-sparing regimens, mainly DRV/r-based monotherapy and dual therapy. ART switch leads to a complete resolution of toxicities in most cases in the short term.

**Figure 1:**
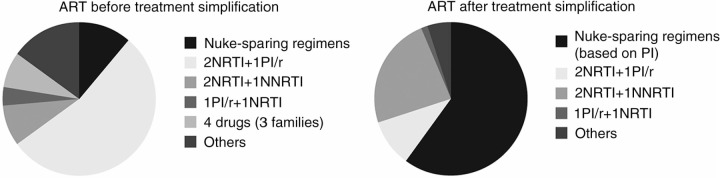
Antiretroviral therapy before and after treatment simplification (N=80)****.

**Figure 2:**
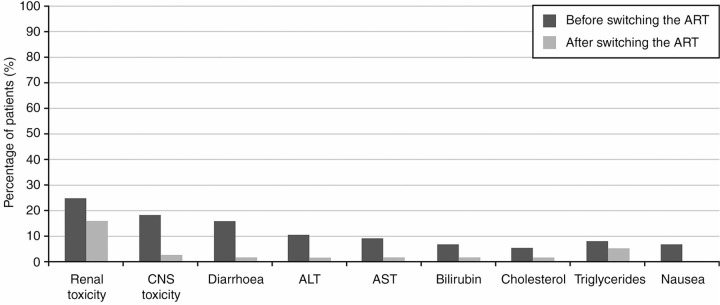
Main toxicities before and after switching the antiretroviral therapy (N=77)****.

